# Supplementary material for: Potential Role of Pig UCP3 in Modulating Adipocyte Browning via the Beta-Adrenergic Receptor Signaling Pathway
Source: Biology (Basel). 2024 Apr 23;13(5):284. doi: 10.3390/biology13050284 (PMC11117546; doi:10.3390/biology13050284)
Supplement: Supplementary file 1 [file biology-13-00284-s001.zip › biology-2952821-supplementary.pdf]

biology

MDPI

Supplementary Materials

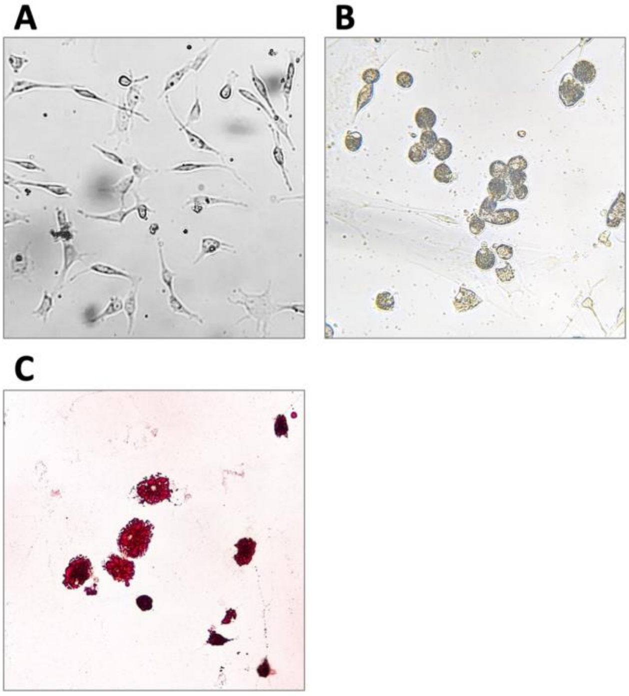

Supplementary Figure S1. Cell morphology changes demonstrating success of the pig adipocyte dedifferentiation-redifferentiation process prior to browning experiments. (A) Dedifferentiation of mature adipocytes to DFAT resulted in a fibroblast-like cell morphology. (B) Redifferentiation of DFAT to adipocytes resulted in a rounded cell morphology. (C) Staining of lipid using Oil Red O.

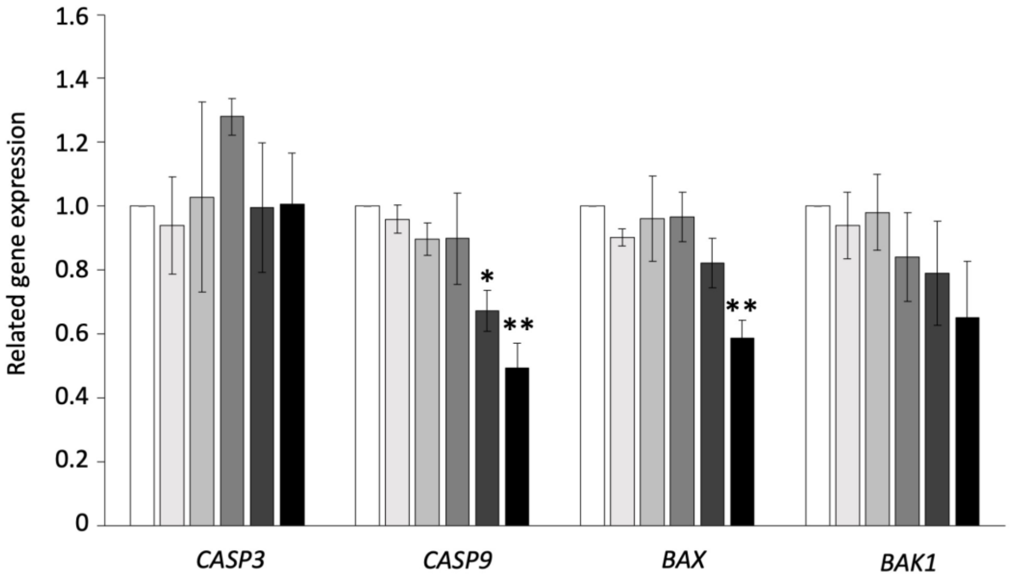

Supplementary Figure S2. Gene expressions related to apoptosis of pig redifferentiated adipocytes after isoproterenol administration. The values of the treatment and control groups were compared. The experiments were conducted 4 times. Each color indicates a concentration of isoproterenol: (□) control, (□) 0.01 μM, (□) 0.1 μM, (■) 1 μM, (■) 10 μM, and (■) 100 μM. The values are shown as mean ± SEM. * $p < 0.05$ and ** $p < 0.01$.

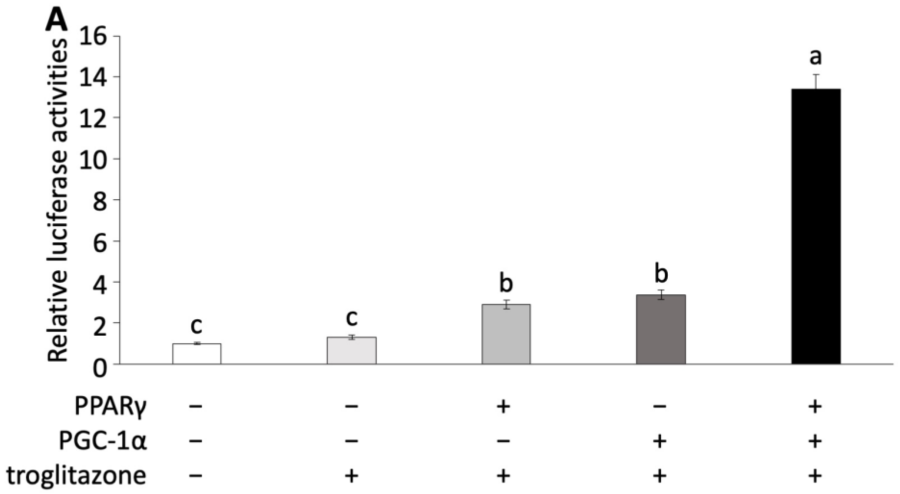

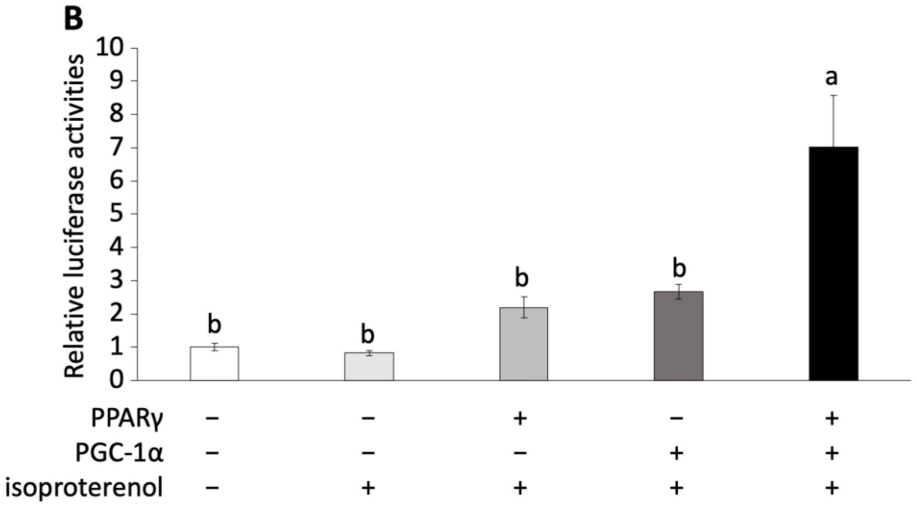

Supplementary Figure S3. Luciferase assay of pig UCP promoter region and alignment analysis. (A) Luciferase assay using Hela cells transfected with *PPARγ* or/and *PGC-1α* with/without 5 μM troglitazone. The experiments were conducted 4 times. (B) Luciferase assay using Hela cells transfected with *PPARγ* or/and *PGC-1α* with 1 μM isoproterenol. The experiments were conducted thrice. The values are shown as mean ± SEM. Significant differences are indicated by different signs.

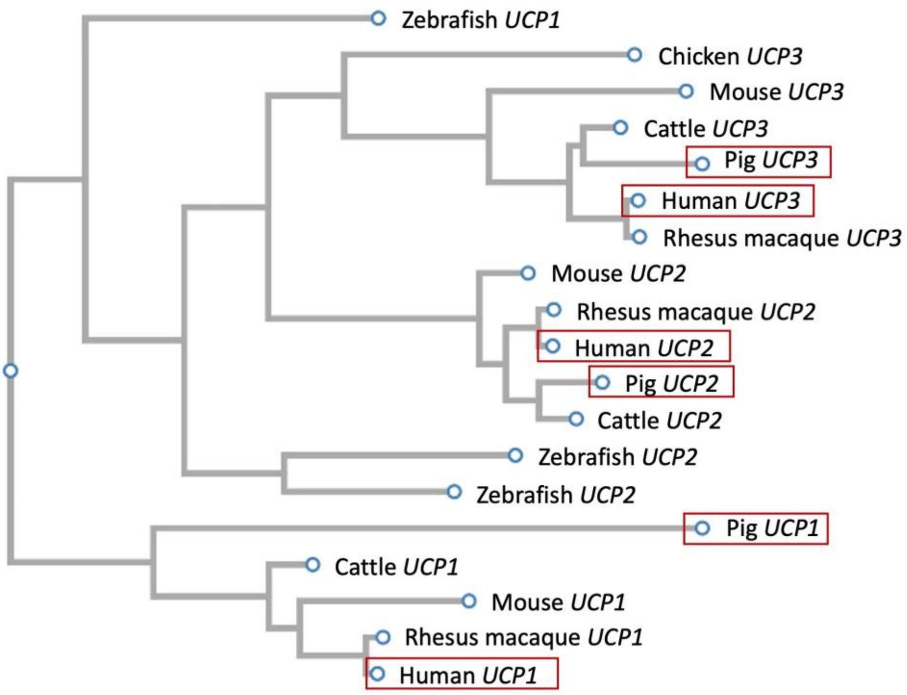

Supplementary Figure S4. Tree diagram of UCP family mRNA sequences in vertebrates. The red-line boxes indicate pig and human genes.

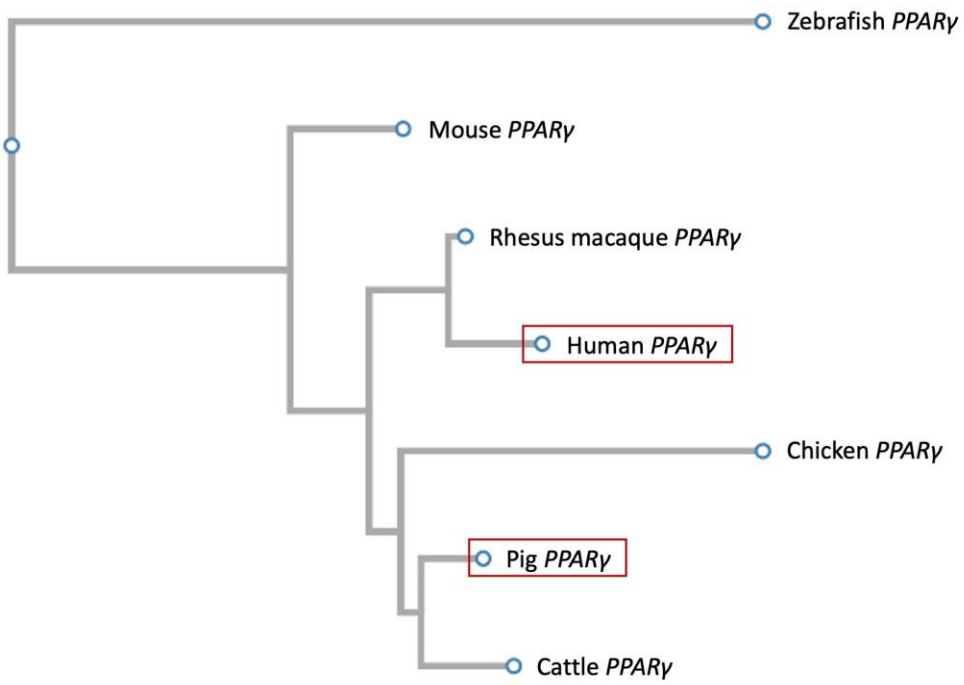

Supplementary Figure S5. Tree diagram of *PPARγ* mRNA sequences in vertebrates. The redline boxes indicate pig and human genes.

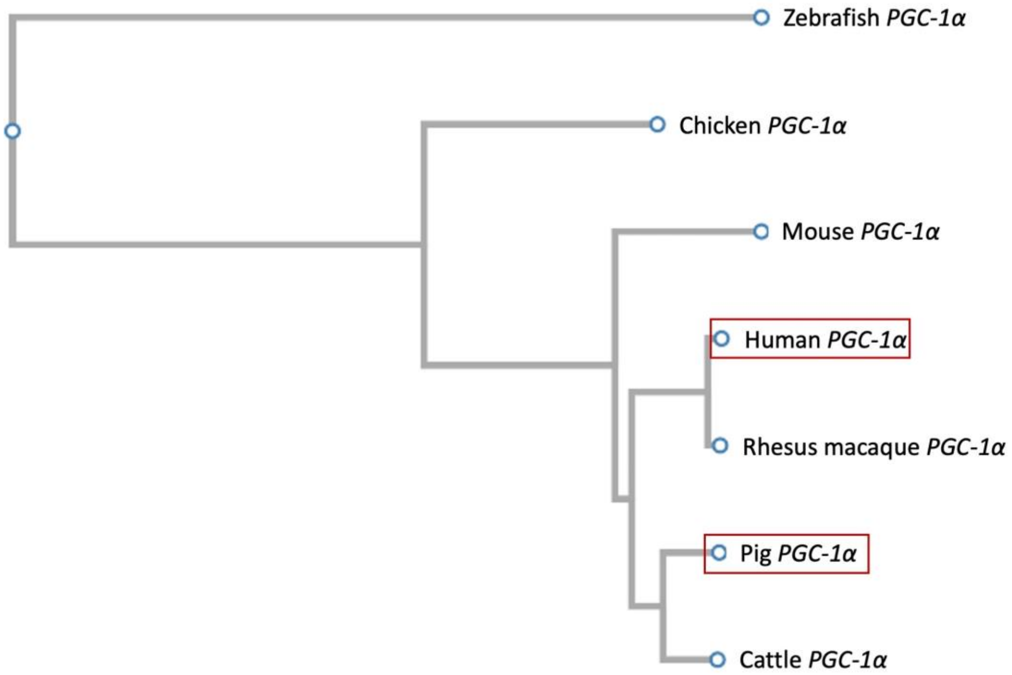

Supplementary Figure S6. Tree diagram of *PGC-1α* mRNA sequences in vertebrates. The redline boxes indicate pig and human genes.

Supplementary Table S1. Primer pairs used in the analysis of gene expressions.

Gene		Primer	Product Length (bp)	Annealing Temperature (°C)	Accession Number
PGC-1α	F	CATGTGCAACCAGGACTCTG	133	60	NM_213963
	R	GCTGTCTGTATCCAAGTCGTTC			
PPARγ	F	AGGACTACCAAAGTGCCATCAAA	142	60	NM_214379
	R	GAGGCTTTATCCCCACAGACAC			
UCP1	F	GCAGGGCAGACAGGTAAGC	134	60	XM_021100543.1
	R	CAAATAGTCCCGCCAAACCA			
UCP2	F	CCTCAGTGTGAGACCTGACGAA	148	60	NM_214289
	R	CTGTGGCCTTGAATCCAACCA			
UCP3	F	ATTCCAGGCCAGCATACACG	165	60	NM_214049
	R	GTCACCATCTCGGCACAGTT			
CS	F	GGCTGACCTTATACCTAAGGAGC	230	60	NM_214276.1
	R	GTTCTTCCCCACCCTTAGCC			
CASP3	F	TGTGGGATTGAGACGGACAG	131	60	NM_214131.1
	R	GATCCGTCCTTTGAATTTCGCC			
CASP9	F	CCAGTGGACATTGGTTCTGGA	247	60	XM_003127618.4
	R	TGGCAGTCAGGTTGCACTTC			
BAX	F	GCCTCAGGATGCATCTACCAA	170	60	XM_003127290.5
	R	AGTTGAAGTTGCCGTCAGCA			
BAK1	F	CTAGAACCTAGCAGCACCATGG	156	60	XM_021098603.1
	R	GGAGGCGATCTTGGTGAAGTAC			
ACTB	F	AGATCAAGATCATCGCGCCTC	159	60	DQ845171.1
	R	AGTCCGCCTAGAAGCATTTGC			

Supplementary Table S2. Alignment scores of thermogenesis genes among mammals.

	**Pig Human**	**Pig Mouse**	**Pig Cattle**	**Human Mouse**	**Human Cattle**	**Mouse Cattle**
PPARγ	85	84	86	84	78	76
PGC-1α	91	90	95	86	90	88
UCP1	30	29	33	70	80	72
UCP2	84	79	86	83	84	80
UCP3	73	52	82	68	86	76

Alignment scores were calculated using mRNA sequences of *PPARγ*, *PGC-1α*, *UCP1*, *UCP2*, and *UCP3* in pigs, humans, mice, and cattle. The heights of the alignment scores indicate the homology of mRNA between two species.

Supplementary Table S3. Alignment scores of the promoter regions in the UCP family among mammals.

	Pig<br>Human	Pig<br>Mouse	Pig<br>Cattle	Human<br>Mouse	Human<br>Cattle	Mouse<br>Cattle
UCP1	53	6	66	18	46	5
UCP2	5	3	5	22	53	3
UCP3	23	18	25	21	60	19

Alignment score calculations in the promoter region used the sequence of 1,000 bp upstream from the transcriptional start site in *UCP1*, *UCP2*, and *UCP3*.
